# The relation between serum levels of interleukin 10 and interferon-gamma with oral candidiasis in type 2 diabetes mellitus patients

**DOI:** 10.1186/s12902-022-01217-x

**Published:** 2022-11-28

**Authors:** Atefe Halimi, Nazanin Mortazavi, Ali Memarian, Maryam Zahedi, Farhad Niknejad, Ahmad Sohrabi, Shakiba Javadian Sarraf

**Affiliations:** 1 Dental Research Center, Golestan University of Medical Sciences, Gorgan, Iran; 2 Department of Oral and Maxillofacial Medicine, School of Dentistry, Golestan University of Medical Sciences, PO Box 4916953363, Gorgan, Iran; 3 Stem Cell Research Center, Golestan University of Medical Sciences, Gorgan, Iran; 4 Department of Immunology, Faculty of Medicine, Golestan University of Medical Sciences, Gorgan, Iran; 5 Department of Internal Medicine, Endocrinology and Metabolic Disorders, Clinical Research Development Unit (CRDU), Sayad Shirazi Hospital, Golestan University of Medical Sciences, Gorgan, Iran; 6 Laboratory Sciences Research Center, Golestan University of Medical Sciences, Gorgan, Iran; 7 Cancer Control Research Center, Cancer Control Foundation, Iran University of Medical Sciences, Tehran, Iran

**Keywords:** Interferon-gamma, Interleukin-10, Oral candidiasis, Type 2 Diabetes Mellitus

## Abstract

**Background:**

Type 2 Diabetes mellitus (T2DM) is one of the most common endocrine diseases that weakens the immune system. *Candida albicans*, is part of the natural oral flora and increases in cases of compromised immune systems. The exact cause of the increased prevalence of candidiasis in patients with T2DM is still unclear. The study aimed to correlate serum interleukin 10 (IL-10) and interferon-gamma cytokines (IFN-γ) with oral candidiasis in T2DM.

**Methods:**

In this case–control study, 81 patients with T2DM and 41 non-diabetic individuals aged 30 to 70 years participated. Demographic information, a Blood sample (for blood glucose and cytokine tests), and an oral cotton swab sample from each individual were obtained. The samples were then incubated in a Sabroud dextrose agar medium. Colony growth was calculated and the type of yeast species in individuals with oral candidiasis was identified by culture in CHROMagar *Candida* medium. IL-10 and IFN-γ were measured by ELISA kit and the data were analyzed using SPSS-18.

**Results:**

An overall of 122 participants comprised 73.77% females and 26.22% males. An increase in interleukin-10 by 40% and a decrease in IFN-γ by 6% can increase oral candidiasis prevalence among diabetic patients. *Candida albicans* was the most prevalent *Candida* species (spp.) in the diabetic and non-diabetic groups. The presence of oral candidiasis was not associated with HbA1c or FBS levels in both groups.

**Conclusion:**

In the diabetic population, an increase in IL-10 or a decrease in IFN-γ may be associated with an increased risk of oral candidiasis.

## Introduction

Diabetes mellitus (DM) is a chronic metabolic and degenerative disorder that is characterized by chronic hyperglycemia resulting from defects in insulin action due to insulin resistance, insulin secretion, or both [1]. In 2019, approximately 463 million adults (20–79 years) were living with diabetes; by 2045 this will rise to 700 million [2]. Due to elevated serum glucose levels and their bad effect on immune system function, diabetic patients are more prone to opportunistic infections such as candidiasis [3]. One of the most common types of candidiasis infection is the oral type [4]. *Candida albicans* is part of the normal oral cavity flora and around 30% to 50% of people carry this organism [5]. Numerous studies have shown that the prevalence of *Candida* in diabetic patients is significantly higher than in healthy individuals [6–9]. In a recent study, the prevalence of *Candida* was 70% in diabetic patients and 30% in non-diabetic individuals [10]. The host conditions most likely to facilitate the colonization and subsequent infection of *Candida* include yeast adhesion to the epithelial surface, glucose accumulation in the saliva, decreased salivary flow, microvascular degeneration, and impaired neutrophil Candidacidal activity. Generalized immunosuppression alters the balance of host yeast by favoring the transition of *Candida* species from commensal to pathogenic [11].

It has been suggested that type 2 DM (T2DM) represents manifestations of the inflammatory host response that are orchestrated by the production of pro-and anti-inflammatory cytokines [12]. Studies indicate that patients with T2DM, have an increase in IFN-γ levels and a decrease in IL-10 levels [12–14]. In this regard, IL-10 is an anti-inflammatory cytokine that plays a critical role in preventing the development of autoimmune diseases [13]. IL-10 dysregulation is associated with enhanced immunopathology as well as an increased risk of developing multiple autoimmune diseases [14]. In addition, polymorphisms in the IL-10 gene increase diabetes risk [12, 15, 16]. On the other hand, IFN-γ as a pro-inflammatory factor has an essential role in T2DM pathogenesis [17]. It also serves as a key component of immunity against fungal diseases like Candidiasis [18, 19].

Despite the higher level of IFN-γ in patients with T2DM, *candida* infections are more common in these patients than healthy individuals and the underlying reasons has yet to be uncovered [6]. This study aimed to investigate the association between IL-10 and IFN-γ serum levels in patients with oral Candidiasis and T2DM. This could support the clarification of principal immuno-pathogenesis mechanisms for candidiasis in T2DM patients.

## Methods

A total of 81 diabetic outpatients were studied at the Gorgan referral Diabetes Clinic. Diabetes mellitus was diagnosed based on the 2020 Diagnostic Criteria of the American Diabetes Association. The Control group included 41 healthy non-diabetic individuals. Case and control groups were matched based on sex and age. The systemic disorders (such as any underlying diseases affecting the immune system, rheumatic diseases, diabetic kidney disease, infectious diseases, recent hospitalization) that could result in candidiasis, use of dentures, antibiotics, corticosteroid therapy, immunosuppressive or inhaled drugs within the previous 4 weeks, smoking, and xerostomia were excluded. The Ethics Committee of Golestan University of Medical Sciences approved the research protocol (IR.GOUMS.REC.1397.178).

Participants were asked to provide demographic information and blood samples were taken after 8 to 10 h of overnight fasting. A volume of 5 ml whole blood was taken from all subjects for detection of Hemoglobin A1c (HbA1c), Fasting Blood Sugar (FBS), IFN-γ and IL-10 levels. HbA1c and FBS were measured in Deziani laboratory by routine capillary electrophoresis and enzymatic methods, respectively. The serum level of IFN-γ and IL-10 were determined using commercially available ELISA kit (Biolegend, CA, USA) and according to the manufacturer’s instructions. All samples were assayed in triplicates, and the results were reported as picograms per milliliter (pg/mL) [20].

Following normal saline rinse of the mouth, two sterile swabs of the tongue were obtained. The first swap was cultured on sabrodextrose agar medium (Conda S.A., Madrid. Spain). Incubation of the culture media (37 °C) was followed by the measurement of growth and number of colonies at 24–48 h (CFU / mm^2^). The diabetic and non-diabetic participants were divided into two groups of with and without candidiasis, based on the culture medium colonies in their subjects. The reference ranges for healthy commensal carriages detected by the swab method were 0–5 CFU/swab, so people with CFU < 5 were classified as non-candidiasis and CFU ≥ 5 as candidiasis group [21].

The second swap of the candidiasis group was cultured on chromium-agar medium (CHROMagar, France) to determine the type of *Candida* species. In chromium agar culture medium, *Candida albicans*, *Candida tropicalis*, and *Candida glabrata* are easily distinguished by their color and morphology. *Candida albicans* produces colonies with varying shades of green, *Candida tropicalis* produces colonies with a blue-gray color, and *Candida glabrata* produces pink colonies. The other colors produced by *Candida*, such as purple and yellow, are classified under the group of other types of *Candida* [22]. The relationship between blood glucose indices (FBS, HbA1c), IL-10, and IFN-γ and the presence or absence of candidiasis were also evaluated in the study groups.

### Statistical analysis

Data normality was assessed by the Shapiro–Wilk test. In case of normality, the T-student test was applied. The non-parametric tests of Mann–Whitney and logistic regression were used to determine the relationships when the data were abnormal. Statistical significance was set at *P* < 0.05. Statistical analyses were performed using the SPSS software version 18 (SPSS, Chicago, IL, USA).

## Results

The present study included 122 individuals aged 30 to 70 years, represented by means, standard deviations shown in Table 1. Of these, 90 were women (73.77%) and 32 were men (26.22%). Of 81 subjects in the patient group, 60 were women (74.07%) and 21 were men (25.92%), and among the 41 participants in the control group, 30 were women (73.17%) and 11 men (26.82%).

**Table 1:** Data summaries in study groups

Groups	N	Candidiasis N (%)		Sex		Age Mean (SD)	FBS Mean (SD)	HbA1c Mean (SD)
				Male N (%)	Female N (%)			
Diabetic	81	+	42 (51.85%)	10 (23.80%)	32 (76.19%)	52.29 (6.947)	166 (62.42)	8.5 (2.07)
		-	39 (48.15%)	11 (28.21%)	28 (71.79%)	51.23 (8.549)	177 (59.13)	8.4 (1.74)
Non-diabetic	41	+	13 (31.70%)	2 (15.38%)	11 (84.62%)	55.85 (6.581)	101 (6.79)	5.8 (0.25)
		-	28 (68.29%)	9 (32.14%)	19 (67.86%)	47.36 (8.807)	95 (10.67)	5.6 (0.27)

Candidiasis was found in 51.85% of the diabetic patients and 31.70% of the non-diabetic individuals. Therefore, an increased risk of oral candidiasis can be associated with diabetes (*P*. value = 0.035). A significant relation between HbA1c and FBS and the development of oral candidiasis was not found in the diabetic or non-diabetic groups in the current study (Table 1). *Candida albicans* had the highest prevalence in both the diabetic and non-diabetic groups with 45% and 69%, respectively.

*Candida tropicalis* was the least common type with a 4% prevalence in the diabetic group. The *Glabratai* species were not detected in the non-diabetic group, and other types of *Candida* accounted for 7% (Table 2).

**Table 2:** Frequency of candida types in study groups

Groups	Candida Type			
	*C. Albicans*	*C. Glabrata*	*C. Tropicalis*	*Other*
Diabetic	21 (45%)	7 (15%)	2 (4%)	16 (34%)
Non-diabetic	9 (69%)	0 (0%)	3 (23%)	1 (7%)
Total	30 (52%)	7 (11%)	5 (8%)	17 (29%)

The mean IL-10 level in positive candidiasis samples was significantly higher than that in negative samples in both diabetic and non-diabetic groups. IL-10 levels were significantly higher in the non-diabetic group compared to the diabetic group in samples negative for candidiasis (Table 3).

**Table 3:** Mean (SD) of IL-10 in study groups

Groups	Candidiasis +	Candidiasis -	Total	*P*. Value
Diabetic	22.60 (22.27)	5.79 (3.98)	14.51 (18.25)	< 0.001 *
Non-diabetic	33.62 (28.93)	16.94 (20.20)	22.23 (24.26)	0.008 *
Total	25.21 (24.19)	10.45 (14.38)	17.10 (20.69)	< 0.001 *
*P*. Value	0.052	0.006 *	0.035 *	

Positive samples of candidiasis had significantly lower levels of IFN-γ than negative samples in both cases (*P*. value < 0.001) and control groups (*P*. value = 0.036) (Table 4).

**Table 4:** Mean (SD) of IFN-γ in study groups

Groups	Candidiasis +	Candidiasis -	Total	*P*. Value
Diabetic	24.25 (18.16)	90.34 (60.05)	56.07 (54.64)	< 0.001*
Non-diabetic	57.79 (118.51)	99.06 (99.34)	85.98 (106.08)	0.036 *
Total	32.18 (59.82)	93.99 (78.30)	66.12 (76.78)	< 0.001 *
*P*. Value	0.565	0.517	0.484	

**Fig. 1:**
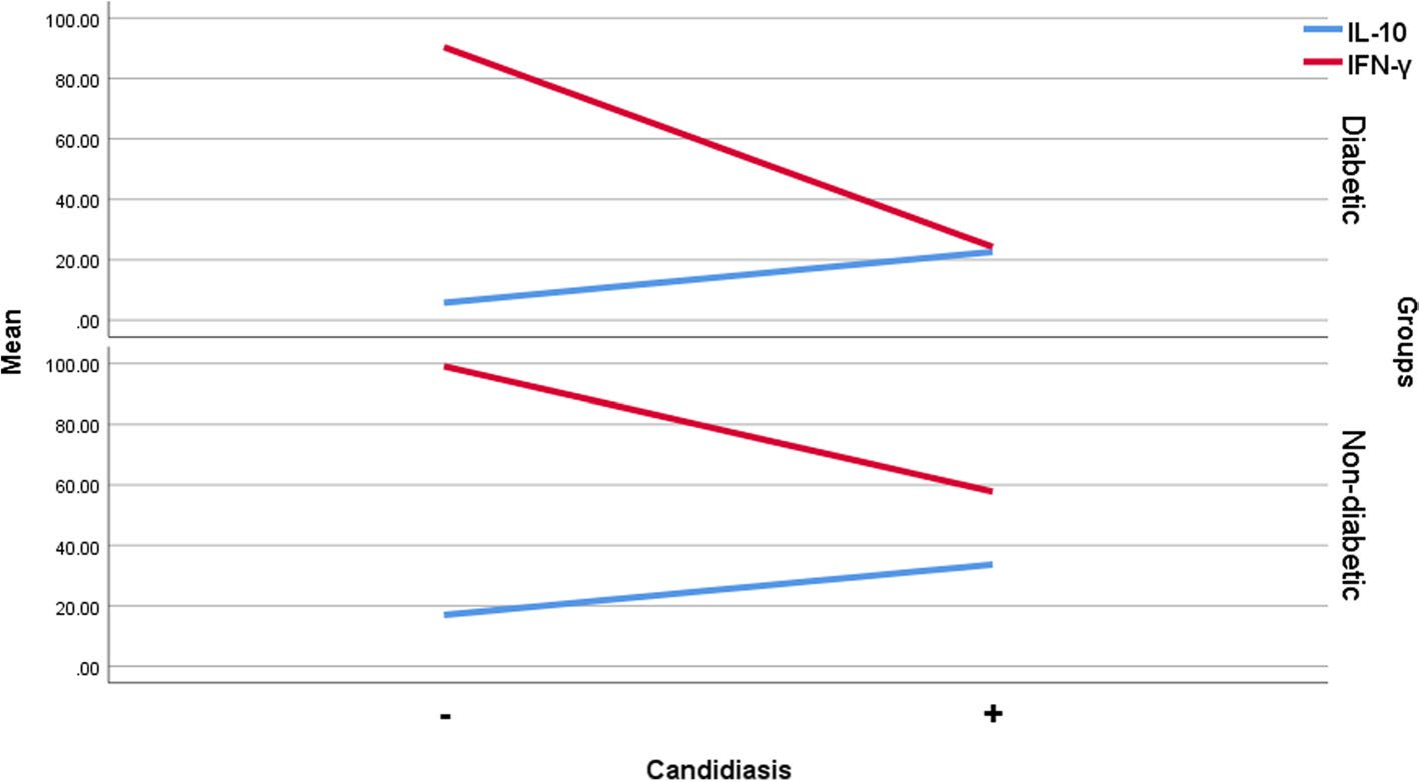
Line plot of IL-10 and IFN-γ means in study groups

According to Fig. 1, an increase in IL-10 and a decrease in IFN-γ are seen in people with oral candidiasis. Due to the dichotomous response variable (candidiasis positive or negative), multivariable logistic regression was used to determine the relationship between IL-10, IFN-γ and the development of candidiasis by controlling the HbA1c. In response to an increase in IL-10, candidiasis prevalence increased by about 40% (OR = 1.40) and (*P*. value ˂ 0 / 001) in diabetic patients. Decreased IFN-γ (OR = 0.94 and *P*. value ˂ 0.001) also increased the risk of candidiasis by 6% in the diabetic group (Table 5).

**Table 5:** Logistic regression models result

Groups			EXP(β) OR	95% CI		Wald	*P*-Value
**Diabetic**	IL-10 Model	IL-10	1.402	1.203	1.633	18.788	< 0.001
		HbA1c^a^	0.784	0.548	1.123	1.763	0.184
	IFN-γ Model	IFN-γ	0.942	0.917	0.968	18.603	< 0.001
		HbA1c^a^	0.852	0.618	1.172	0.970	0.325
**Non-diabetic**	IL-10 Model	IL-10	1.029	0.998	1.062	3.330	0.068
		HbA1c^a^	42.579	1.382	1312.097	4.600	0.032
	IFN-γ Model	IFN-γ	0.995	0.987	1.004	1.261	0.262
		HbA1c^a^	34.498	1.461	814.378	4.819	0.028

^a^HbA1c was used as an independent variable for IL-10 and IFN-γ in the logistic regression models

Neither interleukin 10 nor interferon gamma were significantly related to diabetes mellitus duration (*P*. value = 0.267 and 0.298, respectively. Moreover, no significant relationship was found between age and sex and the development of oral candidiasis.

## Discussion

Following an increase in blood glucose levels and a decrease in immune system activity in patients with diabetes mellitus, these patients are prone to opportunistic infections [23]. Candidiasis is one of the most common oral fungal diseases in healthy people or people with weakened immune systems, such as diabetic patients [3].

IL-10 is an inflammatory cytokine that has an inhibitory effect on autoimmune diseases [13]. The association between T2DM and IL-10 has been studied in several articles, and IL-10 levels in T2DM are lower than those in controls [12].

According to the results of the present study, with a rise in IL-10, the probability of developing oral candidiasis in the diabetic group increases by about 40%. A previous observation from Roilides suggests that IL-10 affects the host PMN phagocytes that are involved in fungal defense, inhibiting PMN and fungi phagocyte activity, thus increasing fungal infection risk [24].

Rani M et al. [25] showed that the IL-10 levels in diabetic patients with oral candidiasis were significantly higher compared with healthy controls. The diabetic group had more oral-pharyngeal candidiasis, and the increase in IL-10 in the diabetic group indicates that the immune system is highly susceptible to chronic disease. In light of the inhibition of fungal phagocytosis by IL-10, it is justified to speculate that the immune system tends to cause chronic progressive disease.

However, the results of the present study contrast with the results of the de Oliveira et al. [26] study, which found low or undetectable IL-10 levels in patients with oral candidiasis.

Rani M. [25] mentioned the levels of IL-10 are generally higher in diabetic patients with oral candidiasis, but the levels are lower when *Candida albicans* antigens are applied as peripheral blood stimulants. The present study measured IL-10 using centrifuged blood serum obtained from patients (in vivo) rather than peripheral blood stimulated with *Candida* antigen (in vitro).

As stated by Yaghini et al. [27], the level of IL-10 in diabetic patients is significantly lower than in the control group. Also in the present study, the level of IL-10 in diabetic patients was lower than in the non-diabetic group.

IFN-γ is important in the regulation of cellular immunity and is effective in the pathogenesis of diabetes and autoimmunity, as it stimulates multiple branches of the immune system [28–30]. A systemic immune response is initiated as *Candida* albicans infects epithelial cells, which increases the amount of IFN-γ produced [31]. Consequently, this host defense may be crucial for controlling *Candida* albicans at the oral site and preventing its spread [26]. Low serum levels of IFN-γ are likely due to diminished production of the cytokine, which might contribute to *Candida* infection [32]. With a decrease in IFN-γ, the chance of developing oral candidiasis increases by 6% [12]. Szkaradkiewicz et al. [32], also found that IFN-γ levels were significantly lower in people with chronic oral candidiasis than healthy individuals.

Elevated salivary glucose is one of the main risk factors for oral candidiasis in diabetic patients [3]. The present study found that people with T2DM have a higher prevalence of oral candidiasis. The role of *Candida* yeasts as a cause of oral candidiasis in diabetic patients was shown by several studies [3, 8, 33–35]. Nevertheless, Bremenkamp R. et al. [36] found no correlation between diabetes and oral candidiasis.

Our results indicate that there is no significant correlation between FBS and HbA1c with the development of oral candidiasis in the diabetic and non-diabetic groups. Zomorodian et al. [3] also found that despite the higher prevalence of candidiasis in diabetic patients, there was no association between HbA1c levels and candidiasis, and this result was confirmed by Belazi M et al. [8] and Kumar BV et al. [34] as well. On the contrary, Shenoy MP et al. found that there is a significant relationship between FBS and HbA1c and oral candidiasis. The Sheony study included patients with type 1 and type 2 diabetes, and the sampling method was oral rinsing rather than swap sampling [37]. Differences in administration methods and patient groups may be responsible for the different outcomes.

The normal oral flora of 73 of the total subjects (59.8%) contained *Candida*, including 65.4 percent of the case group and 48.8 percent of the control group. Zomorodian et al. [3] mentioned that 67.8% of the diabetic group and 50.4% of the control group are carriers of *Candida*. Kumar BV. et al. [34] reported this ratio as 68.52% in the diabetic group and 27% in the control group. In this study, 42 patients in the diabetic group (51%) and 13 patients in the healthy group (31%) had oral candidiasis. Rodrigues CF et al. [11] expressed this percentage in the diabetic group at 68%, and in the healthy group at 27%, while Alzarea et al. [10] expressed it in the patient group at 70%, and in the healthy group at 30%.

A second swap cultured on CHROMagar *Candida* medium identified *Candida albicans* as the most prevalent *Candida* spp. (52%), followed by Other *Candida* spp. (29%), *Candida Glabrata* (11%), and *Candida Tropicalis* (8%). Khadka S et al. [22] reported the most predominant species as *Candida albicans* 64.7%¸ followed by other *Candida* species (15/6%), *Candida tropicalis* 11.7%¸ and *Candida glabrata* 7.8%.

## Conclusion

Study results indicate that there is a significant difference between diabetic and non-diabetic groups regarding susceptibility to oral candidiasis. However, no significant relationship was found between blood glucose indices and the development of oral candidiasis in participants with or without diabetes.

The likelihood of developing oral candidiasis increases as the level of IFN-γ decreases and the level of IL-10 increases.

## Data Availability

The datasets generated and/or analyzed during the current study are not publicly available due to confidentiality of information but are available from the corresponding author on reasonable request.
